# The Effect of Intermittent Fasting on Appetite: A Systematic Review and Meta-Analysis

**DOI:** 10.3390/nu15112604

**Published:** 2023-06-01

**Authors:** Rebecca L. Elsworth, Angelica Monge, Rachel Perry, Elanor C. Hinton, Annika N. Flynn, Alex Whitmarsh, Julian P. Hamilton-Shield, Natalia S. Lawrence, Jeffrey M. Brunstrom

**Affiliations:** 1 Nutrition and Behaviour Unit, School of Psychological Science, University of Bristol, Bristol BS8 1TU, UK; am.monge.11@gmail.com (A.M.); annika.flynn@bristol.ac.uk (A.N.F.); jeff.brunstrom@bristol.ac.uk (J.M.B.); 2 Bristol Heart Institute and Bristol Trials Centre, Bristol Medical School, University of Bristol, Bristol BS8 1NU, UK; rachel.perry@bristol.ac.uk; 3 NIHR Bristol Biomedical Research Centre, University Hospitals Bristol and Weston NHS Foundation Trust and University of Bristol, Bristol BS8 2BN, UK; elanor.hinton@bristol.ac.uk (E.C.H.); j.p.h.shield@bristol.ac.uk (J.P.H.-S.); 4 Population Health Sciences, Bristol Medical School, University of Bristol, Bristol BS8 2PS, UK; alex.whitmarsh@bristol.ac.uk; 5 Department of Psychology, University of Exeter, Exeter EX4 4QG, UK; natalia.lawrence@exeter.ac.uk

**Keywords:** intermittent fasting, appetite, time-restricted eating, alternate day fasting, 5:2 dieting, hunger, fullness

## Abstract

Previously, narrative reviews have considered the effects of intermittent fasting on appetite. One suggestion is that intermittent fasting attenuates an increase in appetite that typically accompanies weight loss. Here, we conducted the first systematic review and meta-analysis to quantify the effects of intermittent fasting on appetite, when compared to a continuous energy restriction intervention. Five electronic databases and trial registers were searched in February 2021 and February 2022. Abstracts (N = 2800) were screened and 17 randomized controlled trials (RCTs), consisting of a variety of intermittent fasting regimes, met our inclusion criteria. The total number of participants allocated to interventions was 1111 and all RCTs were judged as having either some concerns or a high risk of bias (Cochrane RoB 2.0 tool). Random effects meta-analyses were conducted on change-from-baseline appetite ratings. There was no clear evidence that intermittent fasting affected hunger (WMD = −3.03; 95% CI [−8.13, 2.08]; *p* = 0.25; N = 13), fullness (WMD = 3.11; 95% CI [−1.46, 7.69]; *p* = 0.18; N = 10), desire to eat (WMD = −3.89; 95% CI [−12.62, 4.83]; *p* = 0.38; N = 6), or prospective food consumption (WMD = −2.82; 95% CI [−3.87, 9.03]; *p* = 0.43; N = 5), differently to continuous energy restriction interventions. Our results suggest that intermittent fasting does not mitigate an increase in our drive to eat that is often associated with continuous energy restriction.

## 1. Introduction

Intermittent fasting is an increasingly popular diet that involves alternating periods of energy restriction with periods of unrestricted energy intake [1]. Intermittent fasting has been found to produce equivalent weight loss to continuous energy restriction interventions [2,3,4] and there are physiological health benefits [5], e.g., improvements in cardiometabolic risk factors [6,7] and glucose metabolism [8]. Moreover, traditional, continuous restriction requires strict adherence to a diet, with no opportunity for flexibility. For this reason, individuals who follow an intermittent fasting regime may find it easier to achieve sustained weight loss [9]. 

Intermittent fasting regimes are often categorized into one of three types [10]: (i) alternate-day fasting (ADF), (ii) 5:2 dieting, and (iii) time-restricted eating (TRE) [11]. ADF involves alternating between a day of total food abstinence and a day of unrestricted eating [12]. The 5:2 diet involves limiting energy intake to 500 calories in women and 600 calories in men, for two non-consecutive days per week, with unrestricted eating for the rest of the week [13]. TRE is slightly different, as it involves following the same routine every day, where you eat within a certain window of hours and fast in the remaining hours [14], for example, the 16:8 diet, where eating is only allowed within an 8 h window each day [15]. 

Among other factors, appetite may play an important role in adherence to intermittent fasting diets. Appetite is your motivation to eat food [16] and it encompasses feelings such as hunger, fullness, and desire to eat. Notably, intermittent fasting could lead to an individual feeling hungrier than usual which could result in them breaking the fast (i.e., eating during the fasting period) and, in turn, this might promote dietary disinhibition [17]. In some cases, fasting could result in individuals eating before starting the fast, in anticipation of hunger [17]. Despite sometimes being reported as a secondary outcome measure in randomized controlled trials (RCTs), the effect of intermittent fasting on appetite is unclear. Previous reviews suggest that appetite is reduced following intermittent fasting [9,18,19]. More specifically, Seimon et al. [18] suggested that intermittent fasting may attenuate adaptive physiological responses that typically increase the ‘drive to eat’ when undergoing long-term continuous energy restriction [20]. This was corroborated by their finding that in six of ten clinical trials, appetite either decreased or it did not change significantly, following an intermittent fasting intervention. More recently, a review by Liu et al. [9] described that participants’ hunger decreased, and fullness increased, after intermittent fasting interventions when compared to baseline appetite ratings. However, they also reported on four studies that found no significant differences in appetite between intermittent fasting and continuous restriction interventions. Hoddy et al. [19] proposed that ADF may curb appetite over time, but highlighted that the mechanisms behind this are uncertain. 

Here, we conducted the first systematic review and meta-analysis to quantitatively assess the effects of an intermittent fasting intervention on appetite. The primary aim was to directly compare the effects of intermittent fasting and continuous energy restriction interventions, which provided the opportunity to isolate the effects of fasting beyond mere calorie restriction. The secondary aims were to explore the effect of intermittent fasting on body weight, energy intake, eating behavior, and physical activity, when compared to continuous energy restriction interventions, and to assess adherence and dropout rates in both intervention groups.

## 2. Materials and Methods

This review was pre-registered on the International Prospective Register of Systematic Reviews (PROSPERO) database (registration ID: CRD42021245146). In addition, a protocol was written following guidance from the Preferred Reporting Items for Systematic Reviews and Meta-Analyses (PRISMA-P) statement and checklist [21]. This was uploaded to Open Science Framework (URL: https://osf.io/cs8g6/ accessed on 25 April 2023) and we deviated slightly from the protocol by assessing dropout as absolute values (i.e., counts) rather than percentages for the meta-analysis. 

The review was conducted in accordance with the Preferred Reporting Items for Systematic Reviews and Meta-Analyses (PRISMA) statement [22], and the PRISMA 2020 checklist is included in Appendix A.

Published and unpublished randomized controlled trials (RCTs) were eligible for inclusion in the systematic review. There were no limitations on publication date or language. Eligibility criteria were defined using the Participants, Intervention, Comparator, and Outcomes structure [23].

- Participants: Humans of any age and any BMI.
- Intervention: Intermittent fasting interventions of any type (e.g., alternate day fasting, time-restricted eating, 5:2 diet) and any duration.
- Control/comparator: Continuous energy restriction intervention.
- Outcomes: To be included in the review, the RCT must have measured the primary outcome of appetite, e.g., visual-analogue scales of hunger, fullness, desire to eat, and prospective food consumption (PFC). Where measured, secondary outcomes were also included in the review: body weight (kg), energy intake (kcal/day), eating behavior questionnaire scores (e.g., Three-Factor Eating Questionnaire), physical activity, adherence to interventions (%), and dropout.

A systematic three-phased search was carried out. The first phase consisted of running an initial search on MEDLINE, PsycINFO, and EMBASE via OvidSP. Titles, abstracts, and index terms were analyzed and this informed the finalized search. The second phase consisted of running the finalized search on the following databases (MEDLINE and PreMEDLINE (OvidSP) (1950 to 18 February 2022), PsychINFOEMBASE Classic + EMBASE (OvidSP) (1974 to 18 February 2022), PsychINFO (1806 to 18 February 2022), ISI Web of Science: Science Citation Index Expanded (SCIEXPANDED) (1900 to 18 February 2022), ISI Web of Science: Conference Proceedings Citation Index-Science (CPCI-S) (1990 to 18 February 2022), Scopus, and trial registers (NICE, ClinicalTrials.gov, Cochrane Central Register of Controlled Trials). Grey literature was searched via OpenGrey and unpublished studies were sought by contacting experts in the field. The third phase consisted of checking for additional studies through reference lists of included papers. The final search was run (19 February 2021) and updated (18 February 2022). The search strategy consisted of intermittent fasting terms combined with the Cochrane highly sensitive search strategy for identifying randomized controlled trials [24]. The search strategy for MEDLINE (via OvidSP) is included in Appendix B and this was adapted to run on each database accordingly. 

Duplicates were removed using Endnote X9 [25] and abstract and full-text screening was carried out on Rayyan, a web and mobile app for systematic reviews [26]. All titles and abstracts were screened for eligibility by two independent reviewers (R.L.E.; A.M.; A.N.F.; J.M.B.). Of these, full texts of potential papers for inclusion were retrieved and assessed against pre-defined inclusion criteria by two independent reviewers (R.L.E.; A.M.; A.N.F.). Reasons for exclusion were recorded and any inconsistencies were discussed and resolved.

Data from included papers were extracted by two independent reviewers (R.L.E.; A.M.; E.C.H.) using a data extraction form that was adapted throughout the data extraction process. The final data extraction form is included in Appendix C. For RCTs covered in multiple publications, we used the report that presented the most relevant data. Extracted information included sample characteristics (sample size, demographics), details of intermittent fasting and continuous energy restriction interventions (type, duration), primary outcome measures (hunger, fullness, desire to eat, PFC), and secondary outcome measures (body weight, energy intake, eating behavior, physical activity, adherence to interventions, and dropout), as well as information required for a risk of bias assessment. Any inconsistencies were discussed between the two reviewers and resolved.

For each RCT, the risk of bias was assessed by two independent reviewers (R.L.E.; A.N.F.) using Version 2 of the Cochrane risk of bias tool for randomized trials [27]. Any inconsistencies were discussed and resolved. The strength of the overall body of evidence for each outcome domain using the Grading of Recommendations Assessment, Development, and Evaluation (GRADE) methodology [28] was also assessed by two independent reviewers (R.L.E.; R.P.).

All study and participant characteristics were tabulated and summarized. Outcome characteristics were compared to determine whether they were suitable for quantitative synthesis. In cases where an RCT outcome was not able to be included in meta-analyses, data were summarized narratively. Only RCTs that measured appetite using a visual analogue scale were included in the meta-analysis. Visual analogue scales were either on a scale of 10 or 100. In cases where visual analogue scales comprised a 10-point scale, we transformed data by multiplying the means and SDs by 10, so all data were on a scale of 100. For included studies, weighted mean differences with 95% confidence intervals are reported where differences represent differences in change scores (post-intervention appetite minus baseline) between the IF and continuous energy restriction (CER) groups. Heterogeneity between RCTs was assessed using both Chi^2^ and *I*^2^. Random effects meta-analyses were conducted to explore changes in appetite following an intermittent fasting intervention compared to continuous energy restriction, as this considers heterogeneity between RCTs. Meta-analyses were performed and forest plots were produced in Review Manager (RevMan Version 5.4.) [29].

When appetite data were not reported in reports, we requested missing data from the corresponding authors. We provided them with a three-week window to respond and have indicated cases where data were received as author correspondence (Table 3). When required, we back-calculated data following Cochrane guidance [30]. This included calculating standard deviation from standard error or 95% confidence intervals, combining two subgroups into a single group (e.g., high and low weight loss groups), change-from-baseline means, and standard deviations. When imputing SDs for change-from-baseline we assumed a correlation coefficient of 0.5, which is a slightly conservative method [31].

Subgroup analyses consisting of a comparison of 5:2 dieting, ADF, and TRE regimes were performed. We carried out two sensitivity analyses: (1) excluding studies with imputed results, and (2) performing fixed-effects meta-analyses. In our protocol, we stated we would also run sensitivity analysis by including only RCTs classified as low risk of bias, but this was not possible. Funnel plots were used to assess publication bias including for each intermittent fasting regime subgroup separately.

## 3. Results

### 3.1. Study Selection

**Figure 1:**
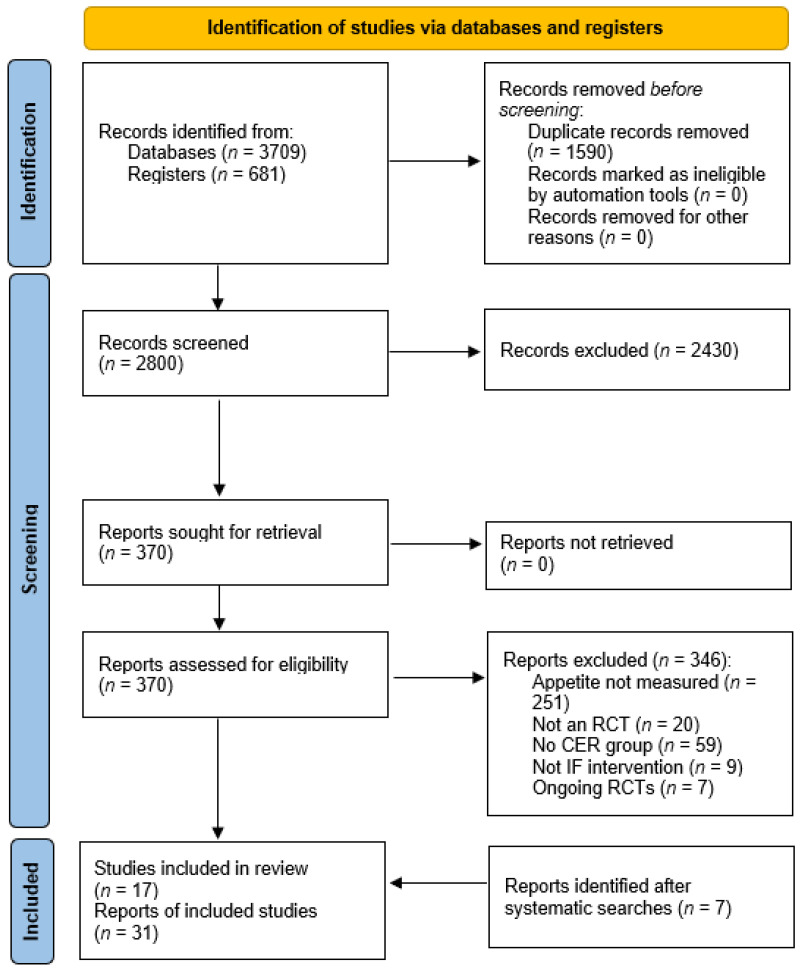
PRISMA 2020 flow diagram [22,32]. CER: continuous energy restriction. IF: intermittent fasting.

A PRISMA flow diagram is presented in Figure 1. Of 4390 records that were identified, 1590 were duplicates and thus the total number of abstracts and titles screened was 2800. This led to 2430 records being excluded and 370 full texts screened for eligibility. A total of 346 full texts were excluded and 7 ongoing trials were identified (Appendix D). No additional RCTs were found through hand searching reference lists of included studies; however, seven additional reports covering the existing included studies were identified. The number of studies included in the review was 17, which corresponds to 31 reports (multiple papers, trial protocols, conference abstracts, etc.). A summary of the quantity and type of reports for each RCT is presented in Appendix E.

### 3.2. Study Characteristics

All included trials were parallel-group RCTs with at least two arms (intermittent fasting and continuous energy restriction). Study characteristics are presented in Table 1. Two studies were pilot RCTs [33,34]. Intermittent fasting interventions comprised a variety of regimes including six ADF [35,36,37,38,39,40], six 5:2 diet [33,34,41,42,43,44], and four TRE [45,46,47,48], with Cai et al. [49] including three arms (ADF, TRE, continuous energy restriction). The duration of trials ranged from 2 weeks to 12 months, with the most common duration being 12 weeks (*n* = 8). Some trial designs consisted of a ‘weight maintenance’ phase following the initial ‘weight loss’ phase. This included Harvie et al. [41], which comprised a 1-month weight-maintenance period after a 3-month period of weight loss, as well as studies by Kroeger et al. [38] and Sundfør et al. [43], which both comprised a 6-month ‘weight maintenance’ phase after a 6-month ‘weight loss’ phase, and an intervention by Hopp et al. [40], which comprised 3 months of weight loss followed by a 9-month weight-maintenance period. For these RCTs, this review uses data from post-intervention scores at the end of the ‘weight loss’ phase.

The total number of participants allocated to the intermittent fasting or continuous energy restriction arm was 1111. However, there was a large range of sample sizes, ranging from 18 to 271 (SD = 59). Participant characteristics are described in Table 2. All studies were carried out in adults, with the majority (*n* = 14) being participants with a BMI ≥ 24 kg/m^2^. Comorbidities included non-alcoholic fatty liver disease (NAFLD) [49], autosomal dominant polycystic kidney disease [40], and excess body fat [42], while another RCT included participants with a family history of breast cancer [41]. Specific demographics included war veterans [34], socially vulnerable/low-income [45], and recreationally active [39].

### 3.3. Risk of Bias

**Figure 2:**
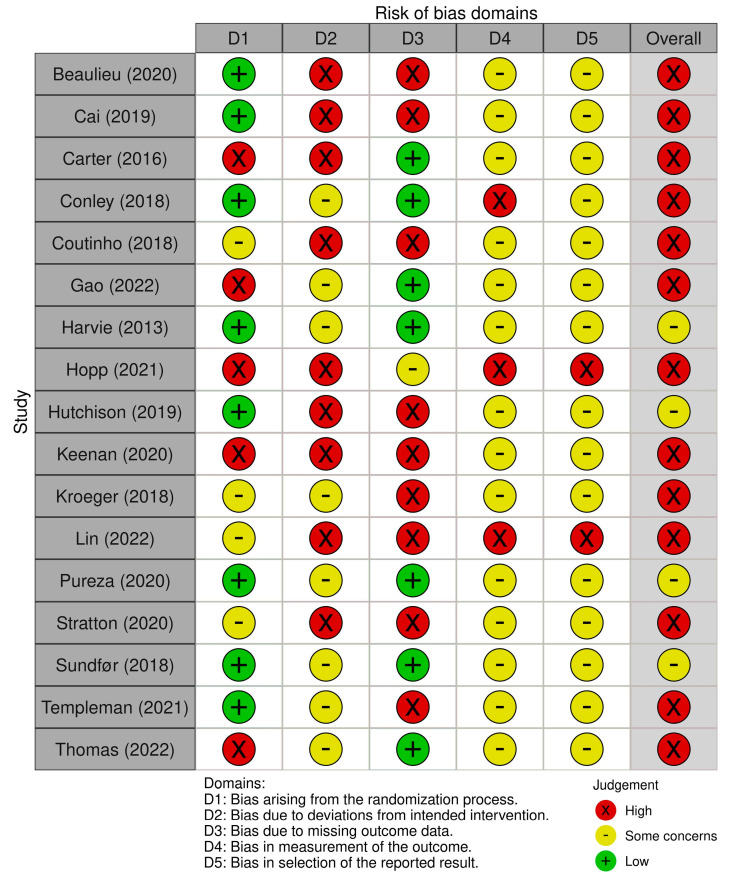
Results from the Cochrane Risk of Bias 2 (RoB 2.0) tool for each study independently. Figure created using the robvis web app [33,34,35,36,37,38,39,40,41,42,43,44,45,46,47,48,49,50].

**Figure 3:**
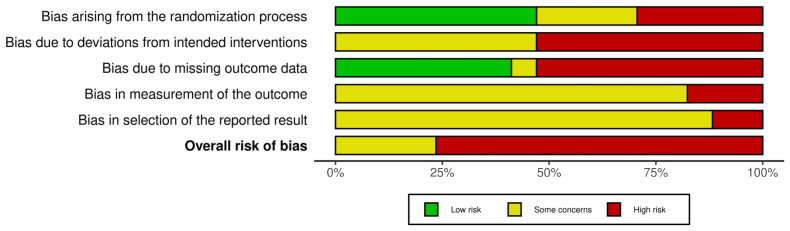
Summary of risk of bias results (*n* = 17). Figure created using the robvis web app [50].

Outcome-based Cochrane risk of bias assessments were conducted on all studies to assess the risk of bias for the measurement of appetite, which was our primary outcome. No RCTs had a low risk of bias, four had a medium risk of bias and thirteen had a high risk of bias. Results for each individual RCT are presented in Figure 2 and a summary of results is presented in Figure 3.

### 3.4. Primary Outcomes

Details of appetite outcomes measured are presented in Table 3. All included RCTs measured appetite in some way; however, measurement of this was variable across studies. The time points at which appetite was measured depended on the duration of the RCT. Mostly, appetite was measured at baseline and then again in the final week of the intervention, or the week after. In some cases, appetite was not measured at baseline, and instead measured when the intervention had already commenced and then in the final week [34,43], or measured more frequently such as weekly [46] or daily [42]. Thirteen of the studies used visual analogue scales, one used Likert scales adapted from a previous visual analogue scales [42], and three asked participants what side effects they were experiencing during the interventions [34,40,48]. In the thirteen studies measuring appetite using visual analogue scales, appetite ratings were obtained in the laboratory following an overnight fast; however, in three studies, appetite was assessed outside the laboratory. This was either in the evening [38,41] or during the day [42].

**Figure 4:**
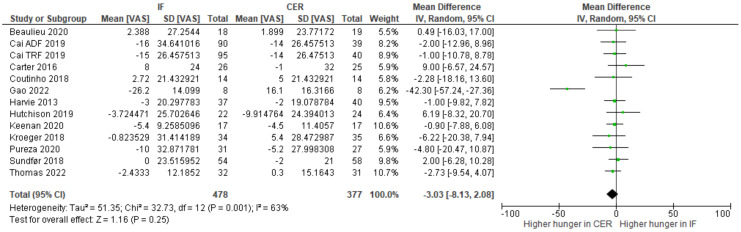
Meta--analysis of change-from-baseline hunger VAS ratings. The forest plot shows effect estimates (green blocks) and 95% confidence intervals (horizontal lines) for each RCT. Larger green blocks indicate a larger weight has been assigned to that RCT. Left of the 0 line shows a finding in favour of intermittent fasting (IF) interventions, whereas right of the 0 line shows a finding in favour of continuous energy restriction (CER) interventions. The diamond at the base of the plot demonstrates the pooled effect estimates and confidence intervals from all RCTs included in the meta-analysis [33,35,36,37,38,41,42,43,44,45,47,49].

**Figure 5:**
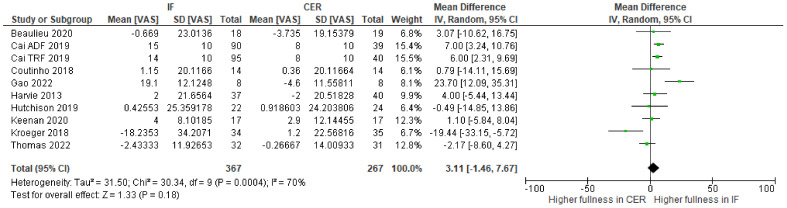
Meta-analysis of change-from-baseline fullness VAS ratings. The forest plot shows effect estimates (green blocks) and 95% confidence intervals (horizontal lines) for each RCT. Larger green blocks indicate a larger weight has been assigned to that RCT. Right of the 0 line shows a finding in favour of intermittent fasting (IF) interventions, whereas left of the 0 line shows a finding in favour of continuous energy restriction (CER) interventions. The diamond at the base of the plot demonstrates the pooled effect estimates and confidence intervals from all RCTs included in the meta-analysis [35,36,37,38,41,42,44,47,49].

**Figure 6:**
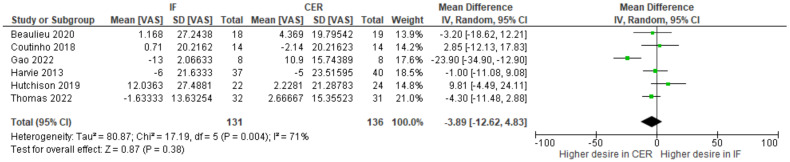
Metaanalysis of change-from-baseline desire to eat VAS ratings. The forest plot shows effect estimates (green blocks) and 95% confidence intervals (horizontal lines) for each RCT. Larger green blocks indicate a larger weight has been assigned to that RCT. Left of the 0 line shows a finding in favour of intermittent fasting (IF) interventions, whereas right of the 0 line shows a finding in favour of continuous energy restriction (CER) interventions. The diamond at the base of the plot demonstrates the pooled effect estimates and confidence intervals from all RCTs included in the meta-analysis [35,36,37,41,44,47].

**Figure 7:**
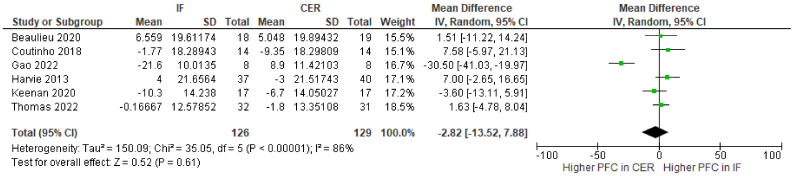
Meta-analysis of change-from-baseline prospective food consumption (PFC) VAS ratings. The forest plot shows effect estimates (green blocks) and 95% confidence intervals (horizontal lines) for each RCT. Larger green blocks indicate a larger weight has been assigned to that RCT. Left of the 0 line shows a finding in favour of intermittent fasting (IF) interventions, whereas right of the 0 line shows a finding in favour of continuous energy restriction (CER) interventions. The diamond at the base of the plot demonstrates the pooled effect estimates and confidence intervals from all RCTs included in the meta-analysis [35,36,41,42,44,47].

Change-from-baseline meta-analyses provided no clear evidence that intermittent fasting interventions affect hunger (Figure 4), fullness (Figure 5), desire to eat (Figure 6), or prospective food consumption (Figure 7), differently to continuous energy restriction interventions.

We also ran subgroup meta-analyses to assess the effects of the different intermittent fasting regimes, when compared to continuous energy restriction on appetite. These meta-analyses showed that when analyzed separately, ADF, TRE, and 5:2 diet interventions did not affect appetite differently from continuous energy restriction interventions.

### 3.5. Secondary Outcomes

**Figure 8:**
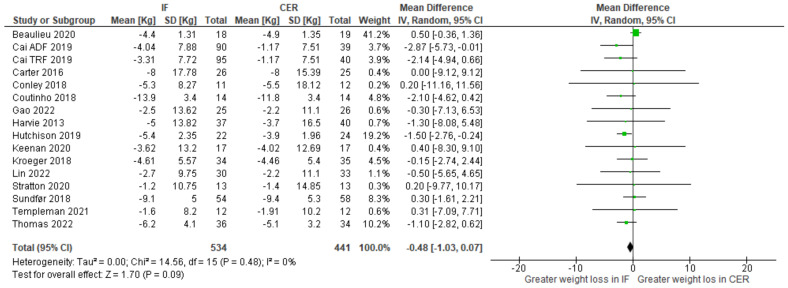
Meta-analysis of change-from-baseline weight (kg). The forest plot shows effect estimates (green blocks) and 95% confidence intervals (horizontal lines) for each RCT. Larger green blocks indicate a larger weight has been assigned to that RCT. Left of the 0 line shows a finding in favour of intermittent fasting (IF) interventions, whereas right of the 0 line shows a finding in favour of continuous energy restriction (CER) interventions. The diamond at the base of the plot demonstrates the pooled effect estimates and confidence intervals from all RCTs included in the meta-analysis [33,34,35,36,37,38,39,41,42,43,44,46,47,48,49].

Body weight: change-from-baseline meta-analyses provided no clear evidence that intermittent fasting interventions affect weight loss (kg) differently from continuous energy restriction (Figure 8).

**Figure 9:**
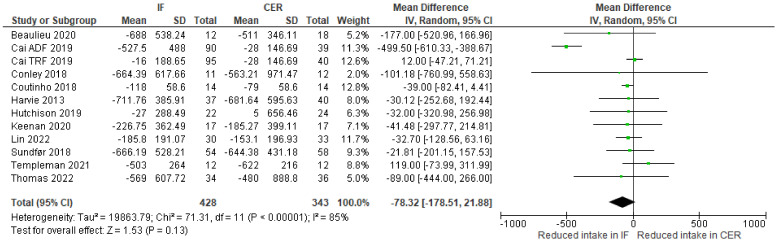
Metaanalysis of change-from-baseline weekly energy intake (kcal). The forest plot shows effect estimates (green blocks) and 95% confidence intervals (horizontal lines) for each RCT. Larger green blocks indicate a larger weight has been assigned to that RCT. Left of the 0 line shows a finding in favour of intermittent fasting (IF) interventions, whereas right of the 0 line shows a finding in favour of continuous energy restriction (CER) interventions. The diamond at the base of the plot demonstrates the pooled effect estimates and confidence intervals from all RCTs included in the meta-analysis [34,35,36,37,39,41,42,43,47,48,49].

Energy intake: change-from-baseline meta-analyses provided no clear evidence that intermittent fasting interventions affect energy intake (kcal) differently from continuous energy restriction (Figure 9).

Eating behavior: Eight RCTs measured eating behaviours (Table 4). Five of these used the Three Factor Eating Questionnaire [35,38,43,46,47], which measures cognitive restraint, disinhibition, and either emotional eating (18-item version) or hunger (51-item version). Due to variability in versions of the Three Factor Eating Questionnaire used, these data were unable to be meta-analyzed. Cognitive restraint increased following the intervention in the intermittent fasting groups in four of the RCTs, whereas in continuous restriction groups, cognitive restraint increased in three of the RCTs but remained the same in one RCT. Susceptibility to hunger decreased in intermittent and continuous restriction groups in the two RCTs measuring hunger, and emotional eating decreased in both groups in the one RCT that measured emotional eating. For intermittent fasting and continuous restriction, disinhibited eating decreased in two RCTs and remained the same in one RCT.

**Figure 10:**
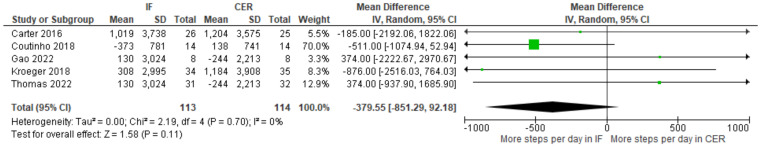
Meta-analysis of change from steps per day. The forest plot shows effect estimates (green blocks) and 95% confidence intervals (horizontal lines) for each RCT. Larger green blocks indicate a larger weight has been assigned to that RCT. Left of the 0 line shows a finding in favour of intermittent fasting (IF) interventions, whereas right of the 0 line shows a finding in favour of continuous energy restriction (CER) interventions. The diamond at the base of the plot demonstrates the pooled effect estimates and confidence intervals from all RCTs included in the meta-analysis [33,36,38,44,47].

Physical activity: Eleven RCTs assessed physical activity. This was measured in a variety of ways including total daily energy expenditure (kcal/day) [35,39], metabolic equivalent of task (MET) values [40,41,43,45], or steps per day [33,36,38,44,47]. Change-from-baseline meta-analysis provided no clear evidence that intermittent fasting interventions affected steps per day differently from continuous restriction interventions (Figure 10).

Adherence: Studies measure adherence in a variety of ways including via self-reported energy intake [35,39,49], food diaries [33,34,36,38,41,43,45,46,48], photographs of food eaten [44], perceived difficulty adhering to the diet [37], how complainant with the diet participants felt [42], and self-reported adherence using questionnaires [40,47]. Adherence was either the same between intermittent fasting and continuous energy restriction arms, or greater in the continuous energy restriction group (Table 5).

**Figure 11:**
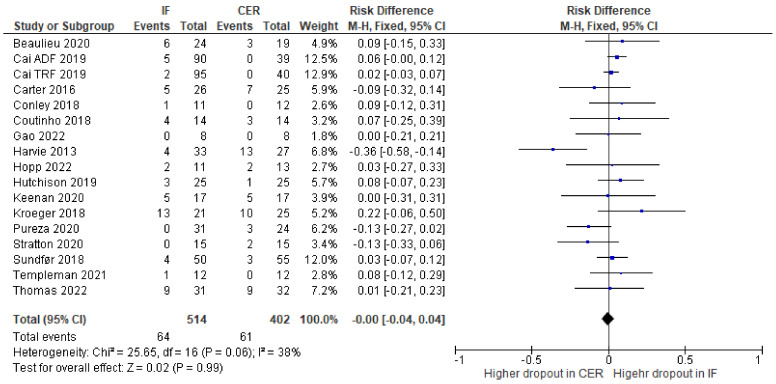
Forest plot for meta-analysis of participant dropout from RCTs. Risk difference reflects difference in dropout between intermittent fasting (IF) interventions and continuous energy restriction (CER) interventions. Larger green blocks indicate a larger weight has been assigned to that RCT. Left of the 0 line shows a finding in favour of intermittent fasting (IF) interventions, whereas right of the 0 line shows a finding in favour of continuous energy restriction (CER) interventions. The diamond at the base of the plot represents the summary result [33,34,35,36,37,38,39,40,41,42,43,44,45,46,47,49].

Dropout: Data on participant dropout were available for all studies except one [48]. Our meta-analysis revealed no evidence that intermittent fasting interventions affected dropout from RCTs differently from continuous energy restriction (Figure 11).

### 3.6. Certainty of the Evidence

We assessed certainty of the evidence of our primary outcome using GRADEpro [28] (Table 6). This revealed that the certainty of evidence was very low for hunger, fullness, desire to eat, and prospective food consumption, indicating that we have little confidence in our effect estimate.

## 4. Discussion

Our meta-analyses provided no clear evidence that intermittent fasting interventions affect hunger, fullness, desire to eat, or prospective food consumption differently than continuous energy restriction interventions. These findings are not in line with existing narrative reviews, which have suggested that intermittent fasting may be associated with an attenuation of the increase in appetite that typically accompanies weight loss [18,19]. One explanation could be that this is a result of differences in analysis approaches. Our review quantitatively compared intermittent fasting interventions to continuous energy restriction interventions, and this approach differs from that used in narrative reviews, where the statistical significance of each individual study is ‘vote counted’ [52]. 

It has been suggested that TRE could allow individuals to maintain the same levels of appetite whilst in a larger calorie deficit, although further research is needed to explore this [53]. By including self-reported energy intake in our meta-analysis, we could assess this across RCTs. We found no evidence that intermittent fasting interventions affected self-reported energy intake differently than continuous energy restriction interventions. 

The systematic methods employed to identify the included studies were stringent, with inclusion of published literature in all languages, alongside grey literature searching, to avoid publication bias. We analyzed results using fixed-effects meta-analysis as a sensitivity analysis. The direction of the effect remained the same for all appetite outcomes; however, we found evidence that intermittent fasting increased fullness, and decreased desire to eat, compared to continuous energy restriction interventions with fixed-effects meta-analysis. Measurement of appetite had either a medium or high risk of bias when assessed using the Cochrane RoB 2.0 tool. This is likely due to the nature of behavioral dietary interventions, where it is often not possible to blind participants or those delivering the intervention. This could result in expectation bias in the intervention group, although this is more pronounced when the control group has no treatment [54], which was not the case in the RCTs included in this review. Moreover, the medium-to-high risk of bias can also be attributed to appetite being assessed using self-report. However, appetite is one’s momentary desire to eat food, and therefore only the participant being asked can report it. Whilst we explored variation in intermittent fasting protocols by conducting subgroup analyses between fasting regimes, we did not consider variability in the degree of continuous energy restriction prescribed. Thus, a concern of the review is that the wide variation in energy restriction protocols and their subsequent effects (e.g., wide variation in daily kcal) could have affected the results of our meta-analysis.

A further concern is that energy restriction diets may have different effects on appetite depending on the time of day that the measurement is taken. Nine of the trials included in the meta-analysis measured appetite at the same time point, namely, in the morning following an overnight fast. However, the RCT by Thomas et al. [47] provides us with the opportunity to explore whether appetite varied throughout the day. They measured appetite before breakfast, before lunch, and before dinner, and found that there was a significant difference in hunger between intermittent fasting and continuous energy restriction groups at lunchtime, but not at breakfast or dinner. This finding indicates that there might be specific times of day when appetite is more susceptible to being modified by a diet. To investigate this further, ecological momentary assessment could be utilized to measure appetite over the course of the whole day. Ecological momentary assessment is an approach that involves repeated sampling of an individual’s experiences in their natural environment [55]. Therefore, ecological momentary assessment could be useful to detect subtle modifications in appetite associated with intermittent fasting interventions. Future research should consider more rigorous measures such as ecological momentary assessment in order to evaluate fluctuations in appetite throughout the day. This approach was suggested for wider evaluation of intermittent fasting interventions in a recent perspective by O’Connor et al., which proposed that ecological momentary assessment could be a useful tool to investigate facilitators and barriers to time-restricted eating adherence [56].

## 5. Conclusions

This review suggests that intermittent fasting interventions are not associated with a reduction in hunger, fullness, desire to eat, or prospective food consumption, when compared to continuous energy restriction interventions. In addition, the review highlights the potential for the use of ecological momentary assessment to investigate fluctuations in appetite throughout the day in future intermittent fasting research. 

## Figures and Tables

**Table 1:** Study characteristics. ADF: alternate day fasting, TRE: time-restricted eating.

First Author (Year)	Country	RCT Design	RCT Duration	Intermittent Fasting Protocol	Continuous Energy Restriction Protocol
Beaulieu (2020) [35]	UK, USA	2 parallel groups	12 weeks	ADF: 25% of daily energy requirements on fast days and ad libitum on feed days	Consume 75% of daily energy requirements each day
Cai (2019) [49]	China	3 parallel groups	12 weeks	ADF: 25% of baseline energy requirements on fast days and ad libitum on feed days. TRE: provided with a meal within an 8-h window and asked to refrain from the consumption of all food or beverages that included energy for the remaining 16 h	Consume 80% of energy needs each day
Carter (2016) [33]	Australia	2 parallel groups (pilot)	12 weeks	5:2 diet: 1670–2500 kJ/day for two days each week and habitual eating for five days each week	7-day continuous energy restriction diet of 5000–6500 kJ/day
Conley (2018) [34]	Australia	2 parallel groups (pilot)	6 months	5:2 diet: daily intake restricted to 600 kcal for two non-consecutive days per week and ad libitum on the remaining five days	Daily 500 kcal reduction from the average requirement
Coutinho (2018) [36]	Norway, Denmark, Australia	2 parallel groups	12 weeks	ADF: 3 non-consecutive days of 550 kcal/day for women and 660 kcal/day for men, and a diet matching energy needs for the remaining four days (≈2118 kcal/day)	Low-calorie diet (≈1410 kcal/day)
Gao (2022) [44]	UK	2 parallel groups	2 weeks	5:2 diet: daily calorie intake restricted to 70% of estimated energy requirements for two non-consecutive days per week, and energy intake of estimated energy requirements for the remaining five days	Daily calorie restriction of 20% from estimated energy requirements
Harvie (2013) [41]	UK, USA	3 parallel groups	12 weeks of weight loss (+4 weeks of weight maintenance)	5:2 diet: 70% energy restriction on two consecutive days per week and meeting estimated energy requirements for the remaining 5 days	25% energy restriction by eating an energy-restricted Mediterranean-type diet
Hopp (2021) [40]	UK	2 parallel groups	3 months of weight loss (+9 months of weight maintenance)	ADF: reduced energy intake to 20% of estimated energy requirements (eaten as a single meal) for three non-consecutive days per week and ate ad libitum for the remaining four days	Daily calorie restriction of approximately 34% of estimated energy requirements
Hutchison (2019) [37]	Australia	4 parallel groups	8 weeks	ADF: 32% of energy requirements at breakfast before a 24-h fast on three non-consecutive weekdays per week and ~100% of energy requirements on the remaining days	Consume 70% of calculated baseline energy requirements
Keenan (2020) [42]	Australia	2 parallel groups	12 weeks	5:2 diet: consume approximately 30% of energy requirements on two non-consecutive days per week, and 100% of energy requirements on the remaining days	Consume approximately 80% of daily energy requirements
Kroeger (2018) [38]	USA	2 parallel groups	6 months (+6 months weight maintenance)	ADF: consume 25% of energy needs on the fast days and 125% of energy needs on the remainder of the days	Consume 75% of energy needs every day
Lin (2022) [48]	Taiwan	2 parallel groups	8 weeks	TRE: 1400 kcal per day consumed within an eight-hour window (10:00–18:00 or 12:00–20:00)	1400 kcal per day with no time restriction
Pureza (2020) [45]	Brazil	2 parallel groups	3 weeks	TRE: 500 to 1000 kcal were subtracted from estimated energy requirements and only eat in a 12-h window	500 to 1000 kcal were subtracted from participants’ estimated energy requirements
Stratton (2020) [46]	USA	2 parallel groups	4 weeks	TRE: 25% caloric deficit and only eat within an 8-h window each day	25% caloric deficit with participants usual daily feeding schedule
Sundfør (2018) [43]	Norway	2 parallel groups	6 months (+6 months weight maintenance)	5:2 diet: consume 400/600 kcal (female/male) on each of two non-consecutive days a week and eat as usual, the remaining five days a week	Reduce energy intake evenly each day so total weekly energy reduction is equivalent in both interventions
Templeman (2021) [39]	UK	3 parallel groups	4 weeks	ADF: alternate between 24-h periods of fasting and eating to 150% of habitual daily energy intake	25% reduction in habitual daily energy intake
Thomas (2022) [47]	USA	2 parallel groups	39 weeks (outcomes measured at 12 weeks)	TRE: 35% daily calorie restriction and only eat within a ten-hour window	35% daily calorie restriction with no instructions on the eating window

**Table 2:** Participant characteristics. ADF: alternate day fasting, TRE: time-restricted eating. Age and BMI data are presented as mean ± SD.

First Author (Year)	Specific Characteristics	N Allocated in Intermittent Fasting/Continuous Energy Restriction	N Analyzed in Intermittent Fasting/Continuous Energy Restriction	Analysis Type	Age (Intermittent Fasting/Continuous Energy Restriction)	Female (Intermittent Fasting/Continuous Energy Restriction)	BMI (Intermittent Fasting/Continuous Energy Restriction)
Beaulieu (2020) [35]	BMI between 25.0 and 34.9 kg/m^2^	24/22	18/19	Completers	36 ± 11/34 ± 9	18/19	29.1 ± 2.2/29.1 ± 2.4
Cai (2019) [49]	NAFLD, BMI > 24 kg/m^2^	95 (ADF) + 97 (TRE)/79	90 (ADF) + 95 (TRE)/79	Completers	35.50 ± 4.417 (ADF), 33.56 ± 6.23 (TRE)/34.54 ± 6.96	60 (ADF), 66 (TRE)/56	26.12 ± 2.21 (ADF), 26.76 ± 1.59 (TRE)/26.34 ± 2.73
Carter (2016) [33]	T2DM with BMI > 27 kg/m^2^	31/32	26/25	Completers	* 61 ± 7.5/62 ± 9.1	* 17/16	* 35 ± 4.8/36 ± 5.2
Conley (2018) [34]	War veterans with BMI ≥ 30 kg/m^2^	12/12	11/12	Completers	68 ± 2.7/ 67.1 ± 3.9	0/0	33.4 ± 1.8/36.2 ± 4.3
Coutinho (2018) [36]	BMI between 30 and 40 kg/m^2^	18/17	14/14	Completers	39.4 ± 11.0/ 39.1 ± 9.0	10/12	35.6 ± 3.2/35.1 ± 4.2
Gao (2022) [44]	BMIBetween 20 and 25 kg/m^2^, and moderately physically active	8/10	8/8	Completers	21 ± 2.8/26 ± 5.7	4/4	21.7 ± 2.3/22.7 ± 1.7
Harvie (2013) [41]	BMI between 24 and 45 kg/m^2^ and a family history of breast cancer	37/40	37/40	Intention to treat	45.6 ± 8.3/ 47.9 ± 7.7	37/40	29.6 ± 4.1/32.2 ± 5.6
Hopp (2021) [40]	Autosomal dominant polycystic kidney disease	13/15	11/13	Intention to treat	46 ± 6/47 ± 12	7/9	34.8 ± 5.1/34.6 ± 5.1
Hutchison (2019) [37]	BMI between 25 and 42 kg/m^2^	25/26	22/24	Completers	* 49 ± 2/51 ± 2	* 25/26	* 32.4 ± 0.8/32.6 ± 1.0
Keenan (2020) [42]	Individuals with a BMI between 22 and 35 kg/m^2^, and excess body fat (>18% for males or >25% for females)	27/27	17/17	Completers	24.8 ± 4.8/23.2 ± 3.9 **	8/9	27 ± 2.7/27.1 ± 2.9 **
Kroeger (2018) [38]	BMI between 25 and 40 kg/m^2^	34/35	34/35	Intention to treat	44 ± 10/ 43 ± 12	30/29	34 ± 4.1/35.6 ± 4.2 **
Lin (2022) [48]	BMI ≥ 24 kg/m^2^	30/33	30/33	Completers	50.1 ± 7.5/54.2 ± 7.9	30/33	25.9 ± 3.7/25.7 ± 3.8
Pureza (2020) [45]	Socially vulnerable/low-income with BMI between 30 and <45 kg/m^2^	31/27	31/27	Intention to treat	31.8 ± 6.9/31 ± 7.1	31/27	33.53 ± 4.8/33.12 ± 3.7
Stratton (2020) [46]	Recreationally active	15/17	13/13	Per protocol	22.9 ± 3.6/22.5 ± 2.2	0/0	Body mass (kg) 82.0 ± 10.6 and height (cm) 178.1 ± 7.3/Body mass (kg) 83.3 ± 15.0 and height (cm) 177.5 ± 8.8
Sundfør (2018) [43]	BMI between 30 and 45 kg/m^2^	54/58	54/58	Intention to treat	49.9 ± 10.1/47.5 ± 11.6	26/30	35.1 ± 3.9/35.3 ± 3.5
Templeman (2021) [39]	BMI between 20.5 and 25.0 kg/m^2^	13/12	12/12	Completers	42 ± 11/45 ± 6	5/7	23.9 ± 2.4/24.0 ± 1.9
Thomas (2022) [47]	BMI between 27 to 45 kg/m^2^	40/41	34/36	Completers	38.3 ± 7.9/37.8 ± 7.8	34/35	34.6 ± 5.8/33.7 ± 5.6

* Baseline characteristics are of participants allocated not participants analyzed, ** Calculated by combining groups.

**Table 3:** Appetite measurement protocols. VAS: visual analogue scale, PFC: prospective food consumption. * Indicates where appetite data were obtained via author correspondence.

First Author (Year)	Primary Outcomes Measured	Timepoint Measured	Appetite Measurement Protocol
Beaulieu (2020) [35]	Hunger, fullness, desire to eat, PFC *	Baseline, week 12	Following an overnight fast, VAS (100 mm) before and after standard breakfast
Cai (2019) [49]	Hunger, fullness, PFC	Baseline, week 4, week 12	VAS (100 mm)
Carter (2016) [33]	Hunger, fullness	Baseline, week 12	Following the overnight fast, VAS
Conley (2018) [34]	Hunger	2 weeks, 3 months, 6 months	‘Any side effects were recorded in individual participant visit notes’
Coutinho (2018) [36]	Hunger, fullness, desire to eat, PFC *	Baseline, week 13	Following overnight fast, VAS (100 mm) before and after standard breakfast
Gao (2022) [44]	Hunger, fullness, desire to eat, PFC *	Baseline, day 7	Following an overnight fast, VAS before and after and standardized liquid breakfast
Harvie (2013) [41]	Hunger, fullness, desire to eat, PFC	Baseline, 1 month, 3 months, 4 months	‘How hungry have you felt over the past day?’ for 3 days, VAS
Hopp (2021) [40]	Hunger	Baseline, 3 months, 12 months	Reported at adverse events
Hutchison (2019) [37]	Hunger, fullness, desire to eat *	Baseline, week 1, week 6	Following overnight fast, VAS (100 mm)
Keenan (2020) [42]	Hunger, fullness *	Daily, from week 1 until week 12	Assessed daily on a mobile phone with a Likert scale (0–10) adapted from VAS
Kroeger (2018) [38]	Hunger, fullness	Baseline, 3 months, 6 months, 9 months, 12 months	VAS (100 mm) before bed for 3 days
Lin (2022) [48]	Hunger	No information	Reported as a side effect
Pureza (2020) [45]	Hunger	Baseline, day 21	Following an overnight fast, VAS (0–10)
Stratton (2020) [46]	Hunger, fullness, desire to eat	Weekly	VAS (0–10), at arrival to a training session
Sundfør (2018) [43]	Hunger	3 months, 6 months, 12 months	Following the overnight fast, VAS (1–10)
Templeman (2021) [39]	Hunger, fullness, desire to eat, PFC	Week 5 (after 4 weeks of monitoring), week 9 (after 4 weeks of intervention)	Following overnight fast, VAS (100 mm)
Thomas (2022) [47]	Hunger, fullness, desire to eat, PFC	Baseline, week 12	Before and after each meal for three days

**Table 4:** Eating behavior measurement and results.

First Author (Year)	Eating Behavior Measure	Timepoints Measured	Findings
Beaulieu (2020) [35]	Three Factor Eating Questionnaire, Binge Eating Scale, Control of Eating Questionnaire, Food Reward, The Leeds Food Preference Questionnaire	Baseline and final week	Dietary restraint increased in both groups. Susceptibility to hunger decreased in both groups. Disinhibited eating decreased more in the continuous restriction than in intermittent fasting.
Gao (2022) [44]	Eating Attitudes Test	At screening	Not reported
Hopp (2021) [40]	Questionnaire on Eating and Weight Patterns-Revised	Baseline, month 3, month 12	Not reported
Kroeger (2018) [38]	Three Factor Eating Questionnaire	Baseline and month 12	There were no significant differences in restraint from baseline to 12 months
Templeman (2021) [39]	Two alternate forced choice tasks	Pre and post intervention	Not reported.
Thomas (2022) [47]	Three Factor Eating Questionnaire	Baseline, week 12, week 39	Dietary restraint increased in both groups similarly from baseline to week 12 and week 12. Disinhibition and susceptibility to hunger did not change-from-baseline to week 12 or 39.
Stratton (2020) [46]	Three Factor Eating Questionnaire	Pre and post intervention	Cognitive restraint increased in the time-restricted eating group but remained the same in the continuous energy restriction group.
Sundfør (2018) [43]	Three Factor Eating Questionnaire	Baseline and month 3	Disinhibited eating and emotional eating reduced in both groups following the interventions. Cognitive restraint increased in both groups, but this increase was greater in the continuous energy restriction group than in the intermittent fasting group.

**Table 5:** Participants’ adherence to interventions.

First Author (Year)	Adherence in Intermittent Fasting Group (%)	Adherence in Continuous Energy Restriction Group (%)
Beaulieu (2020) [35]	83.5	89.2
Conley (2018) [34]	73	75
Coutinho (2018) [36]	78	82
Harvie (2013) [41]	80	80
Stratton (2020) [46]	86.7	86.7

**Table 6:** Summary of the certainty of the evidence, figure created using the GRADEpro GDT app [51]. CI: confidence interval; MD: mean difference. The circles in the certainty column represent the quality of the evidence for one of four grades (very low ⨁◯◯◯, low ⨁⨁◯◯, moderate ⨁⨁⨁◯, high ⨁⨁⨁⨁).

Certainty Assessment							№ of Patients		Effect		Certainty	Importance
№ of Studies	Study Design	Risk of Bias	Inconsistency	Indirectness	Imprecision	Other Considerations	[Intervention]	[Comparison]	Relative (95% CI)	Absolute (95% CI)		
Hunger (change-from-baseline) (assessed with visual analogue scale)												
13	randomized trials	serious	serious	not serious	serious	none	478	377	-	MD 3.03 lower (8.13 lower to 2.08 higher)	⨁◯◯◯ Very low	
Fullness (change-from-baseline) (assessed with visual analogue scales)												
10	randomized trials	serious	serious	not serious	serious	none	367	267	-	MD 3.11 higher (1.46 lower to 7.67 higher)	⨁◯◯◯ Very low	
Desire to eat (change-from-baseline) (assessed with visual analogue scales)												
6	randomized trials	serious	serious	not serious	serious	none	131	136	-	MD 3.89 lower (12.62 lower to 4.83 higher)	⨁◯◯◯ Very low	
Prospective food consumption (change-from-baseline) (assessed with visual analogue scales)												
6	randomized trials	serious	serious	not serious	serious	none	126	129	-	MD 2.82 lower (13.52 lower to 7.88 higher)	⨁◯◯◯ Very low	

## Data Availability

Data sharing not applicable.

## Appendix A

**Table A1:** PRISMA 2020 Main Checklist [22,32]. This comprises 7 sections including a total of 27 items (numbers 1–27), some of which include sub-items (letters a–f).

Topic	No.	Item	Location Where Item Is Reported
TITLE			
Title	1	Identify the report as a systematic review.	1–3
ABSTRACT			
Abstract	2	See the PRISMA 2020 for Abstracts checklist	
INTRODUCTION			
Rationale	3	Describe the rationale for the review in the context of existing knowledge.	51–72
Objectives	4	Provide an explicit statement of the objective(s) or question(s) the review addresses.	71–78
METHODS			
Eligibility criteria	5	Specify the inclusion and exclusion criteria for the review and how studies were grouped for the syntheses.	90–104
Information sources	6	Specify all databases, registers, websites, organisations, reference lists and other sources searched or consulted to identify studies. Specify the date when each source was last searched or consulted.	105–117
Search strategy	7	Present the full search strategies for all databases, registers and websites, including any filters and limits used.	117–120, Appendix B
Selection process	8	Specify the methods used to decide whether a study met the inclusion criteria of the review, including how many reviewers screened each record and each report retrieved, whether they worked independently, and if applicable, details of automation tools used in the process.	121–127
Data collection process	9	Specify the methods used to collect data from reports, including how many reviewers collected data from each report, whether they worked independently, any processes for obtaining or confirming data from study investigators, and if applicable, details of automation tools used in the process.	128–137
Data items	10a	List and define all outcomes for which data were sought. Specify whether all results that were compatible with each outcome domain in each study were sought (e.g., for all measures, time points, analyses), and if not, the methods used to decide which results to collect.	98–104
	10b	List and define all other variables for which data were sought (e.g., participant and intervention characteristics, funding sources). Describe any assumptions made about any missing or unclear information.	128–137
Study risk of bias assessment	11	Specify the methods used to assess risk of bias in the included studies, including details of the tool(s) used, how many reviewers assessed each study and whether they worked independently, and if applicable, details of automation tools used in the process.	138–143
Effect measures	12	Specify for each outcome the effect measure(s) (e.g., risk ratio, mean difference) used in the synthesis or presentation of results.	151–153
Synthesis methods	13a	Describe the processes used to decide which studies were eligible for each synthesis (e.g., tabulating the study intervention characteristics and comparing against the planned groups for each synthesis (item 5)).	144–147
	13b	Describe any methods required to prepare the data for presentation or synthesis, such as handling of missing summary statistics, or data conversions.	159–166
	13c	Describe any methods used to tabulate or visually display results of individual studies and syntheses.	157–158
	13d	Describe any methods used to synthesize results and provide a rationale for the choice(s). If meta-analysis was performed, describe the model(s), method(s) to identify the presence and extent of statistical heterogeneity, and software package(s) used.	147–158
	13e	Describe any methods used to explore possible causes of heterogeneity among study results (e.g., subgroup analysis, meta-regression).	167–168
	13f	Describe any sensitivity analyses conducted to assess robustness of the synthesized results.	168–171
Reporting bias assessment	14	Describe any methods used to assess risk of bias due to missing results in a synthesis (arising from reporting biases).	171–172
Certainty assessment	15	Describe any methods used to assess certainty (or confidence) in the body of evidence for an outcome.	140–143
RESULTS			
Study selection	16a	Describe the results of the search and selection process, from the number of records identified in the search to the number of studies included in the review, ideally using a flow diagram.	174–186
	16b	Cite studies that might appear to meet the inclusion criteria, but which were excluded, and explain why they were excluded.	184
Study characteristics	17	Cite each included study and present its characteristics.	187–221
Risk of bias in studies	18	Present assessments of risk of bias for each included study.	222–232
Results of individual studies	19	For all outcomes, present, for each study: (a) summary statistics for each group (where appropriate) and (b) an effect estimate and its precision (e.g., confidence/credible interval), ideally using structured tables or plots.	233–341
Results of syntheses	20a	For each synthesis, briefly summarise the characteristics and risk of bias among contributing studies.	222–341
	20b	Present results of all statistical syntheses conducted. If meta-analysis was done, present for each the summary estimate and its precision (e.g., confidence/credible interval) and measures of statistical heterogeneity. If comparing groups, describe the direction of the effect.	233–341
	20c	Present results of all investigations of possible causes of heterogeneity among study results.	262–265
	20d	Present results of all sensitivity analyses conducted to assess the robustness of the synthesized results.	338–342
Reporting biases	21	Present assessments of risk of bias due to missing results (arising from reporting biases) for each synthesis assessed.	319–320
Certainty of evidence	22	Present assessments of certainty (or confidence) in the body of evidence for each outcome assessed.	316–320
DISCUSSION			
Discussion	23a	Provide a general interpretation of the results in the context of other evidence.	321–330
	23b	Discuss any limitations of the evidence included in the review.	338–350
	23c	Discuss any limitations of the review processes used.	350–354
	23d	Discuss implications of the results for practice, policy, and future research.	355–371
OTHER INFORMATION			
Registration and protocol	24a	Provide registration information for the review, including register name and registration number, or state that the review was not registered.	80–81
	24b	Indicate where the review protocol can be accessed, or state that a protocol was not prepared.	81–84
	24c	Describe and explain any amendments to information provided at registration or in the protocol.	84–86
Support	25	Describe sources of financial or non-financial support for the review, and the role of the funders or sponsors in the review.	386–391
Competing interests	26	Declare any competing interests of review authors.	397–399
Availability of data, code and other materials	27	Report which of the following are publicly available and where they can be found: template data collection forms; data extracted from included studies; data used for all analyses; analytic code; any other materials used in the review.	Appendix C

## Appendix B. Search Strategy in MEDLINE (via OvidSP)

1. (intermittent adj2 fast*).ti,ab.

2. (intermittent adj2 energy).ti,ab.

3. (intermittent adj2 calorie).ti,ab.

4. (intermittent adj2 restrict*).ti,ab.

5. (intermittent adj2 diet).ti,ab.

6. (alternate day adj2 fast*).ti,ab.

7. (alternate day adj2 diet).ti,ab.

8. time restricted.ti,ab.

9. 5:2 fast*.ti,ab.

10. 5:2 diet.ti,ab.

11. (periodic adj2 fast*).ti,ab.

12. (periodic adj2 diet).ti,ab.

13. 1 or 2 or 3 or 4 or 5 or 6 or 7 or 8 or 9 or 10 or 11 or 12

14. randomized controlled trial.pt.

15. controlled clinical trial.pt.

16. randomized.ab.

17. placebo.ab.

18. drug therapy.fs.

19. randomly.ab.

20. trial.ab.

21. groups.ab.

22. 14 or 15 or 16 or 17 or 18 or 19 or 20 or 21

23. exp animals/ not humans.sh.

24. 22 not 23

25. 13 and 24

## Appendix C

**Table A2:** Data Extraction Form.

Study details	Study number	
	Title	
	Authors	
	Year	
	Form	
	Country	
	Status	
	Funding	
	Conflicts of interest	
	RCT design	
	Trial registration link	
Sample	Total N allocated	
	Analysis type	
	Total N analysed (intention to treat)	
	Total N analysed (completers analysis)	
	Total N analysed (per protocol analysis)	
	Per protocol or completers requirements	
	N allocated	IF
		CER
	N analysed	IF
		CER
	Age (mean, SD)	IF
		CER
	Gender	IF
		CER
	BMI	IF
		CER
	Comorbidities	
	Other demographic information	
Outcome measures	Test day details	
	Hunger	Details
		Timepoint
	Fullness	Details
		Timepoint
	Desire to eat	Details
		Timepoint
	Prospective food consumption	Details
		Timepoint
	Body weight	Details
		Timepoint
	Energy intake	Details
		Timepoint
	Eating behaviour	Details
		Timepoint
	Physical activity	Details
		Timepoint
	Adherence	Details
		Timepoint
IF intervention	Protocol	
	Duration	
	N analysed	
	Hunger (unadjusted mean, SD)	Pre
		Post
		Change score
		Text from paper
	Fullness (unadjusted mean, SD)	Pre
		Post
		Change score
		Text from paper
	Desire to eat (unadjusted mean, SD)	Pre
		Post
		Change score
		Text from paper
	PFC (unadjusted mean, SD)	Pre
		Post
		Change score
		Text from paper
	Body weight (unadjusted mean, SD)	Pre
		Post
		Change score (weight loss)
		Text from paper
	Energy intake (unadjusted mean, SD)	Pre
		Post
		Change score
		Text from paper
	Eating behaviour (unadjusted mean, SD)	Pre
		Post
		Change score
		Text from paper
	Physical activity (unadjusted mean, SD)	Pre
		Post
		Change score
		Text from paper
	Withdrew/ lost to follow up (n)	
	Completed (n)	
	Attrition (%)	
	Adherence (%)	
CER intervention	Protocol	
	Duration	
	N analysed	
	Hunger (unadjusted mean, SD)	Pre
		Post
		Change score
		Text from paper
	Fullness (unadjusted mean, SD)	Pre
		Post
		Change score
		Text from paper
	Desire to eat (unadjusted mean, SD)	Pre
		Post
		Change score
		Text from paper
	PFC (unadjusted mean, SD)	Pre
		Post
		Change score
		Text from paper
	Body weight (unadjusted mean, SD)	Pre
		Post
		Change score (weight loss)
		Text from paper
	Energy intake (unadjusted mean, SD)	Pre
		Post
		Change score
		Text from paper
	Eating behaviour (unadjusted mean, SD)	Pre
		Post
		Change score
		Text from paper
	Physical activity (unadjusted mean, SD)	Pre
		Post
		Change score
		Text from paper
	Withdrew/ lost to follow up (*n*)	
	Completed (*n*)	
	Attrition (%)	
	Adherence (%)	
Training		
Papers from references		

## Appendix D. Ongoing RCTs

https://clinicaltrials.gov/ct2/show/NCT03571048 (accessed on 25 April 2023)

https://clinicaltrials.gov/ct2/show/NCT04138160 (accessed on 25 April 2023)

https://clinicaltrials.gov/ct2/show/NCT04692532 (accessed on 25 April 2023)

https://clinicaltrials.gov/ct2/show/NCT03803072 (accessed on 25 April 2023)

https://clinicaltrials.gov/ct2/show/NCT04327141 (accessed on 25 April 2023)

https://clinicaltrials.gov/ct2/show/NCT03689608 (accessed on 25 April 2023)

https://clinicaltrials.gov/ct2/show/NCT04834687 (accessed on 25 April 2023)

## Appendix E

**Table A3:** RCTs Included in the Review and Their Corresponding Reports. *’s Represents Reports Identified after the Finalized Systematic Search via Hand Searching Reference Lists or Author Correspondence.

First Author (Year)	Papers	Protocol Paper	Conference Abstract	Trial Register	Thesis
Beaulieu (2020) [35]	[35,57]		[58]	[59]	
Cai (2019) [49]	[49]				
Carter (2016) [33]	[33]				
Conley (2018) [34]	[34]				
Coutinho (2018) [36]	[36]			[60]	
Gao (2022) [44]	[44]				
Harvie (2013) [41]	[41]				
Hopp (2021) [40]	[40]				
Hutchison (2019) [37]	[37]			[61]	
Keenan (2020) [42]	[45,62] *		[63] *	[64]	[65] *
Kroeger (2018) [38]	[38]				[66] *
Lin (2022) [48]	[48]				
Pureza (2020) [45]	[45,67]				
Stratton (2020) [46]	[46]				
Sundfør (2018) [43]	[56,68] *				
Templeman (2021) [39]	[39] *	[69]			[70] *
Thomas (2022) [47]	[47]				
